# Microbial Community Evolution Is Significantly Impacted by the Use of Calcium Isosaccharinic Acid as an Analogue for the Products of Alkaline Cellulose Degradation

**DOI:** 10.1371/journal.pone.0165832

**Published:** 2016-11-02

**Authors:** Isaac A. Kyeremeh, Christopher J. Charles, Simon P. Rout, Andrew P. Laws, Paul N. Humphreys

**Affiliations:** Department of Biology, School of Applied Sciences, University of Huddersfield, Huddersfield, West Yorkshire, United Kingdom; Kyungpook National University, REPUBLIC OF KOREA

## Abstract

Diasteriomeric isosaccharinic acid (ISA) is an important consideration within safety assessments for the disposal of the United Kingdoms’ nuclear waste legacy, where it may potentially influence radionuclide migration. Since the intrusion of micro-organisms may occur within a disposal concept, the impact of ISA may be impacted by microbial metabolism. Within the present study we have established two polymicrobial consortia derived from a hyperalkaline soil. Here, α-ISA and a diatereomeric mix of ISAs’ were used as a sole carbon source, reflecting two common substrates appearing within the literature. The metabolism of ISA within these two consortia was similar, where ISA degradation resulted in the acetogenesis and hydrogenotrophic methanogenesis. The chemical data obtained confirm that the diastereomeric nature of ISA is likely to have no impact on its metabolism within alkaline environments. High throughput sequencing of the original soil showed a diverse community which, in the presence of ISA allowed for the dominance the Clostridiales associated taxa with *Clostridium clariflavum* prevalent. Further taxonomic investigation at the genus level showed that there was in fact a significant difference (p = 0.004) between the two community profiles. Our study demonstrates that the selection of carbon substrate is likely to have a significant impact on microbial community composition estimations, which may have implications with respect to a safety assessment of an ILW-GDF.

## Introduction

Microbial activity is likely to affect the performance of the United Kingdoms’ deep geological disposal facility (GDF) concept, particularly when concerning the disposal of intermediate level radioactive wastes (ILW) [1]. In brief, the GDF concept involves the disposal of waste packages within a facility up to 1km below the surface, where the host site geology acts as the first outer barrier to migration [1]. Containers used to store the wastes also act as a physical barrier to radionuclide migration [1]. The waste itself and the repository as a whole will be encapsulated in cement based materials resulting in the generation anoxic, alkaline conditions that retain the radionuclides within the GDF through sorption and precipitation events [1].

Within these evolved conditions, the cellulosic materials that are present within ILW undergo alkaline hydrolysis which results in the formation of a number of cellulose degradation products [2]. The major products of this reaction under simulated repository conditions are the diasteromeric α-isosaccharinic acid (α-ISA) and β-isosaccharinic acid (β-ISA) [2, 3]. ISAs’ have received particular attention due to their ability to form complexes with a number of radionuclides, potentially enhancing their migration through a GDF [4, 5].

Microbial metabolism of these ISAs’ is likely to have an impact on the efficiency of an ILW-GDF, limiting the solubility and migration of radionuclides resulting from ISA complexation. The anoxic, low redox nature of an ILW-GDF coupled to the limited availability of more energetically favourable terminal electron acceptors means that a fermentative microbial metabolism is likely be prevalent [1]. The generation of gas, and associated pressurization may also impact on performance [6, 7]. This has resulted in a number of studies attempting to identify the impact of microbial intrusion and metabolism of ISA’s. The studies of Rout *et al* [8–10] and Charles *et al* [11] adopted the use of a synthetic CDP leachate [12] containing a mixture of both diastereomers of ISA, whilst the work of Bassil *et al* [13] and Kuippers *et al* [14] have chosen α-ISA as carbon source. A number of these studies have used the sediment from the same geographic location [15]. The community and metabolic profiles of these works are however, not directly comparable as a result of variations in basal medium, culturing methods and incubation times.

The aim of the present study was to culture a CDP driven and α-ISA driven consortia established from a single alkaliphilic sediment source. Here, comparisons between the chemical and community profiles of these consortia may indicate the most appropriate carbon source to use within microbiological studies relevant to an ILW-GDF.

## Methods

### Soil sample collection

Soil samples were taken at depth (~10cm) from a hyperalkaline contaminated site at Harpur Hill, Buxton, Derbyshire, UK (53° 14’ 8.4732” N, 1° 55’ 1.2648” W). ISA has already been observed within these soils as a result of the alkaline hydrolysis of organic matter and these soils also contain a diverse population of microorganisms [9]. As an orphaned site, no specific permissions were required to sample and the field studies did not involve endangered or protected species.

### Reactor set-up and chemical analysis

Two cellulose degradation product (CDP) soil reactors were prepared by the addition of the collected soil (5g) to 100mL bottles in duplicate, which was then diluted using 72mL of a previously described anaerobic mineral (pH 9.0) media under a stream of nitrogen [16]. To these reactors, a previously described CDP (8mL) leachate was added [10]. Calcium α-isosaccharinic acid driven soil reactors were prepared via the dilution of 5g of the soil in 80mL of mineral media in duplicate. Ca(α-ISA)_2_ (79.6 mg, prepared as per [17]) was then added to each reactor in duplicate such that the total concentration of ISA in both reactor types was equal. The pH of all the reactors was adjusted to pH 9.0 using sterile 4M NaOH, before the entire volume was flushed with nitrogen for 20 minutes. Reactors wrapped in foil to exclude light before being incubated at 25°C and stirred at 100rpm. In order to employ a batch/feed regimen on the reactors, 8mL of the total volume was removed at the end of 7 days of sampling. This removed volume was then replaced with a 10% solution of CDP diluted in mineral media (CDP reactors) or 7.96 mg of Ca(α-ISA)_2_ diluted in mineral media. The headspace was also de-pressurized and purged with nitrogen at the end of each cycle. This feed cycle was continued on a weekly basis for ten weeks, such that the entire reactor volume had been replaced. During this tenth week, 1.5mL of reactor fluid was taken from each reactor on a daily basis. The pH of the sample was measured before being centrifuged and filtered through a 0.45μm syringe filter unit and frozen for downstream analysis. The total ISA concentration, volatile fatty acid content and headspace gas composition were all determined using methods previously described in Rout et al [10]. pH of samples was measured using a pH meter and calibrated electrodes (Mettler Toledo, UK) and total biomass was estimated using previously described methods [11].

### DNA extraction and Community analysis

Soil sample (~2g) was dissolved in phosphate buffered saline (pH7, 50mL) before being centrifuged at 8000 x g, the pellet was then washed a further three times using PBS with interim centrifugation. Following the final rinse, the pellet was then retained for extraction of DNA. Fluid from the duplicate reactors were pooled and centrifuged at 8000 x g to pellet the cells, DNA was then extracted from these resultant pellets and the prepared soil using the methods of Griffiths *et al* [18]. The V4 region of the 16S rRNA gene was amplified using duel primers 519F (5’CAGCMGCCGCGGTAA’3) and 785R (5’TACNVGGGTATCTAATCC’3) for both bacteria and archaea [19, 20] with the following overhangs 5’ TCGTCGGCAGCGTCAGATGTGTATAAGAGACAG’3 and 5’ GTCTCGTGGGCTCGGAGATGTGTATAAGAGACAG’3, respectively. PCR products were purified using a Qiaquick PCR purification kit (Qiagen, UK) and 16S microbial Community analysis was carried out via a MiSeq platform (Illumina, USA) at 250bp paired ends with chimera detection and removal performed via the UNCHIME algorithm in the Mothur suite [21] (Chunlab, South Korea) before identification using the EzTaxon-e database (http://eztaxon-e.ezbiocloud.net/) [22]. Beta diversity between of the communities using principal component analysis using the vegan package [23] implemented in RStudio 0.99 [24]. Comparative statistical analysis of the taxa observed within the communities were carried out via a Cramér von Mises-type statistic followed by a Monte Carlo test procedure as described by Singleton *et al* [25].

### Nucleotide sequence data

Miseq data has been submitted to Genbank and is available within Bioproject PRJNA325203 under SRA: SRX2037254, SRX2037245 and SRX2037240.

## Results

### Chemical profiles of established consortia

**Fig 1:**
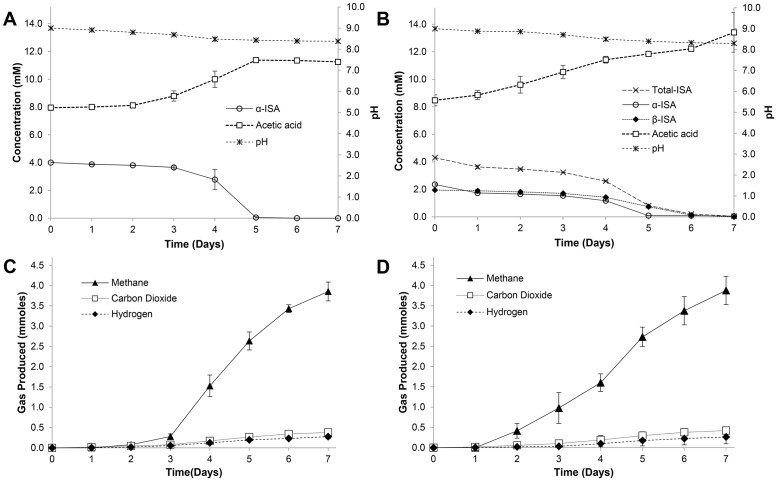
ISA degradation and gas generation in model systems. (A) α-ISA driven system and (B) CDP driven system chemical profiles. Gas generation observed within (C) α-ISA and (D) CDP driven systems (standard deviation shown (n = 2) in all cases).

In the consortia fed solely on α-ISA (Fig 1A), a total removal of ISA occurred within 6 days of incubation. In the CDP fed microcosms the β-diastereomer of ISA was completely removed within 6 days of sampling (Fig 1B), where 3.06 μmoles of α-ISA could still be detected at the end of sampling. The first order rate of ISA degradation across the two systems were determined to be 0.17 ± 0.01 d^-1^ within the α-ISA driven system and 0.18 ± 0.00 d^-1^ within the CDP driven system. In both systems, the removal of ISA coincided with the generation of acetic acid and biomass (Fig A in S1 File). The accumulation of acetic acid occurred in both systems, evidenced by elevated concentrations being observed at day 0. In each case, over the sampling period 0.25 ± 0.00 mmoles of acetic acid were produced in the α-ISA system, with 0.30 ± 0.07 mmoles within the CDP equivalent. pH was not attenuated throughout the sampling process; despite this, only a small decrease in pH was observed within both systems. The generation of gas was also observed within the headspace of each system, where methane, carbon dioxide and hydrogen were evolved. Within the α-ISA driven system (Fig 1C) 3.9mmol ± 0.23 of methane was present in the headspace, with 3.9mmol ± 0.34 present within the headspace of the CDP system (Fig 1D). Methane generation in the α-ISA system lagged behind that in the CDP system by 2 days even though the overall amounts were comparable.

### Microbial community profiling based on 16S rRNA gene comparison

**Fig 2:**
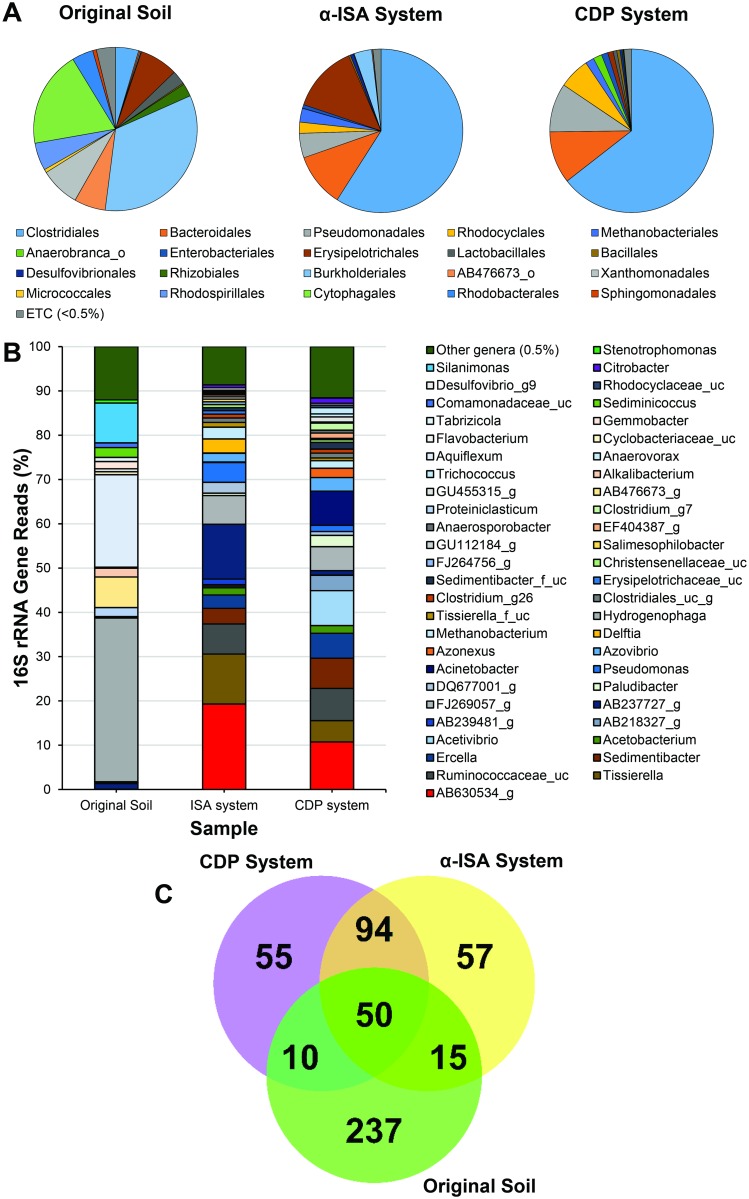
System community profiling. Taxonomic analysis shown at the order (A) and genus (B) classification of the soil, α-ISA and CDP associated communities. Venn diagram of shared and unique genera across the three profiles (C).

Within both the α-ISA and CDP driven systems, taxa associated with the Clostridiales dominated both community profiles, representing 59.0% and 64.5% of the totals respectively (Fig 2A). At the genus level, within both these systems taxa of the genus designation AB630534 were most prevalent, representing 19.3% of the total α-ISA driven system and 10.7% of the CDP driven system (Fig 2B). Clones from this genus all showed greatest sequence homology to *Clostridium clariflavum*, which has been implicated with the generation of acetic acids from carbohydrates [26]. Taxa from the genus *Tissierella* were also prevalent genera of the Clostridiales, representing 11.3% of the α-ISA driven community and 4.8% of the CDP driven community. In both systems, these taxa were showed greatest sequence homology to *Tissierella creatinini*, which has been shown to grow in extremes of pH up to pH 9.1 [27]. A number of taxa within a genus of unclassified Ruminococcaceae were also present within both community profiles, comprising 6.8% and 7.3% of the α-ISA and CDP driven systems respectively. In a similar fashion, taxa from the genera *Sedimentibacter*, *Ercella* and *Acetobacterium* were present in both community profiles representing 1.6, 3.5 and 3.0% (α-ISA driven) and 1.7, 6.8 and 5.6% (CDP driven) of the total reads.

Members of the genus *Acetovibrio* were prevalent within the Clostridiales within the CDP driven system, representing 7.9% of the total community, with only 0.4% of the total reads associated with this genus in the α-ISA driven community. The clones within the *Acetibivrio* showed greatest sequence homology to *Clostridium straminisolvens*, previously associated with cellulolytic processes [28]. Taxa from the genus designation AB218327 were also present in a larger proportion within the CDP driven system compared to the α-ISA driven counterpart, representing 3.5% and 0.3% of the total communities. Here, the clones showed greatest sequence homology to *Anaerotruncus colihominis*, capable of growth up to a pH of 11 and the fermentation of a range of sugars to acetic acid and gas [29]. Although only comprising 1.2% of the total reads, taxa from the genus designation AB239481 were more prevalent within the α-ISA driven system than the CDP driven counterpart, where this genus was representative of 0.04% of the total reads.

Within the α-ISA driven system taxa from the order Erysipelotrichales were the next most prevalent, representing 13.3% of the total community (Fig 2A). Organisms from this order fell within the genus designation AB237727, with clones showing greatest sequence homology to a poorly described species, *Dielma fastidiosa*. Within the CDP driven system, the Erysipelotrichales comprised only 1.1% of the total community, where Bacteroidales associated taxa comprised the second most prevalent order, representing 10.3% of the total. The Bacteroidales were also prevalent within the α-ISA driven system, comprising 10.6% of the total community. At the genus taxonomic classification level, the most prevalent Bacteroidales were of the designation FJ269057, representing 6.5% (α-ISA) and 5.4% (CDP) of the total community profiles. The Bacteroidales were also represented by *Paludibacter* and designation DQ677001 at the genus taxonomic level. Of these two genera, DQ677001 associated taxa were more prevalent within the α-ISA driven system than *Paludibacter*, with the inverse being the case within the CDP driven system profile.

Taxa of the Pseudomonadales also comprised a proportion of the total communities, comprising 4.8% of the α-ISA driven system and 9.6% of the CDP driven system. At the genus classification, the α-ISA driven system was primarily comprised of *Pseudomonas* associated taxa, representing 4.5% of the total community. Within the CDP driven system, *Pseudomonas* associated taxa represented 1.4% of the total community, with *Acinetobacter* associated taxa representing the most prevalent Pseudomonadales (7.7%). Taxa of the order Rhodocyclales were also more prevalent in CDP driven system, representing 6.1% of the total when compared to 2.2% within the α-ISA counterpart. In both systems, the Rhodocyclales were dominated by taxa of the genus *Azovibrio*, with *Azonexus* also being abundant within the CDP driven system. Taxa of the order Burkholderiales represented 3.5% of the α-ISA driven community profile, where these taxa were present within the CDP driven system, but represented only 0.1% of the total community. A significant proportion of this order was represented by taxa of the genus *Delftia*, comprising 3.2% of the total community. Archaeal taxa of the order Methanobacteriales were present within both communities profile, comprising 2.7% of the α-ISA driven system and 1.7% of the CDP-driven system. At the genus taxonomic classification this order was represented almost exclusively by taxa of the genus *Methanobacterium*. The clones within this genus showed greatest sequence homology to *Methanobacterium alcaliphilum*, a hydrogenotrophic methanogen capable of growth up to a pH of 9.9 [30]. Within the CDP driven system 55 unique taxa were present (Fig 2C), with 57 unique genera observed within the α-ISA driven system. Overall, there were 94 shared taxa between these two systems.

The original soil sample obtained contained a diverse population dominated by the order Burkholderiales representing 33.7% of the total community (Fig 2A), of which taxa of the genus *Hydrogenophaga* were observed (Fig 2B). In addition, taxa from the Xanthomonadales, Rhodospirillales, Rhodobacterales, Rhizobiales and Sphingomonadales together represented 20.8% of the total community within the phylum Betaproteobacteria. Taxa associated with the Cytophagales were also abundant in the soil, but absent from either of the ISA driven systems. At the genus level, 237 unique taxa were observed within the soil, where 50 taxa were shared between all three samples (Fig 2C).

## Discussion

The results presented within this study present the first demonstration of methanogenesis from α-ISA as a sole carbon source at pH values relevant to the alkaline disturbed zone of an ILW-GDF. In addition, our study, when coupled to recent findings by Kuippers *et al* [14] and Rout *et al* [8, 9] show that both α-ISA alone and a mixed carbon source as part of CDP are likely to support a methanogenic system within the operational period of such a facility. Irrespective of the source of ISA (single/mixed diastereomers), the degradation rates observed here were similar. This group of findings suggest that the metabolism of both ISA diastereomers occurs through the same metabolic pathway, however they also conflict with previous studies which suggest that α-ISA is not degradable without the presence of a terminal electron acceptor [13].

The diversity of genera present in the soil obtained from the hyperalkaline contaminated site suggested, as other authors have noted, that the microorganisms present within the sediments represent a diverse pool of metabolic potential. The use of ISAs’ as a substrate for growth within a fermentative system appears to have selected for Clostridiales associated taxa. The prevalence of these taxa was unsurprising considering their strong affiliation with anaerobic fermentation of carbohydrates and correlation with previous studies [31, 32]. Across the two systems there were 94 shared taxa, of which 28 of these were of the Clostridiales. Despite similarities, quantitative comparisons between the two community profiles indicated that there was a significant difference (p = 0.004, Fig B in S1 File) between the two systems. This was further confirmed by beta diversity analysis by PCA, which showed that the two system communities were different from each other and from the original soil sample (Fig C in S1 File).

The differences between the two community profiles is likely to be as a result of the more complex mixture of carbonaceous compounds present within CDP. The method used for the generation of CDP has been shown previously produce a liquor containing a number of organic acids of which ISAs represent >70% of the total organic carbon [8]. There was a significant proportion of organic carbon left uncharacterized within this study as a result of the detection limits of the equipment used, previous research has shown that the number of the organic acids generated from the alkaline hydrolysis of cellulose can be extensive [33]. This is perhaps reflected within increased abundance of *Acinetobacter* sp within the CDP driven system, where previous studies have indicated their ability to grow under anaerobic conditions utilizing a wide variety of short chain organic acids [34, 35].

The generation of methane was almost certainly linked to the detection of Methanobacteriales associated taxa, in particular species of *Methanobacterium alcaliphilum*. This reflects previous studies in which the soil at Harpur Hill has yielded methanogens of this species [9, 11]. Since the volumes of methane generated within each systems was similar, ISA is likely the main substrate for methanogenenis. The other carbon sources present within CDP are therefore likely to not be metabolized to methane, and may indeed be recalcitrant to these organisms. The recalcitrance of some of the components of CDP has previously been observed [8, 10]. Within this study, acetic acid accumulated to 63.7% (α-ISA) and 61.9% (CDP) of theoretical levels, suggesting that a degree of acetate oxidation was occurring. The removal of acetate is not strictly limited to members of the Archaea, since previous authors have noted the potential for syntrophic interaction between acetic acid oxidizing bacteria and hydrogenotrophic methanogens [36].

## Conclusion

Overall the results of this study are the first direct comparison of ISA substrates in a single or mixed diastereomeric composition. No significant impact was observed with respect to the degradation pathway of ISA. Data generated from this, and previous studies are therefore applicable to mathematical modelling approaches towards radiological safety assessments for an ILW-GDF concept. The variations observed within community profiles suggest that carbon source selection may be of importance with respect to species estimation following GDF site selection from the incumbent soil/groundwater microbiome.

## Supporting Information

S1 File Fig A in S1 File. Biomass generated within α-ISA (closed diamonds) and CDP (open squares) driven systems. Fig B in S1 File. Cramér von Mises-type statistic followed by a Monte Carlo test procedure comparing α-ISA driven community X with CDP driven community Y, comparing X with Y (A) and Y with X (B). Fig C in S1 File. Beta diversity analysis using Principal Component Analysis of original soil, ISA and CDP systems.
(DOCX)
