# Supplementary material for: Microbial Community Evolution Is Significantly Impacted by the Use of Calcium Isosaccharinic Acid as an Analogue for the Products of Alkaline Cellulose Degradation
Source: PLoS One. 2016 Nov 2;11(11):e0165832. doi: 10.1371/journal.pone.0165832 (PMC5091744; doi:10.1371/journal.pone.0165832)
Supplement: S1 File — Fig A in S1 File. Biomass generated within α-ISA (closed diamonds) and CDP (open squares) driven systems. Fig B in S1 File. Cramér von Mises-type statistic followed by a Monte Carlo test procedure comparing α-ISA driven community X with CDP driven community Y, comparing X with Y (A) and Y with X (B). Fig C in S1 File. Beta diversity analysis using Principal Component Analysis of original soil, ISA and CDP systems. (DOCX) [file pone.0165832.s001.docx]

# Supporting information

Fig A. Biomass generated within α-ISA (closed diamonds) and CDP (open squares) driven systems.


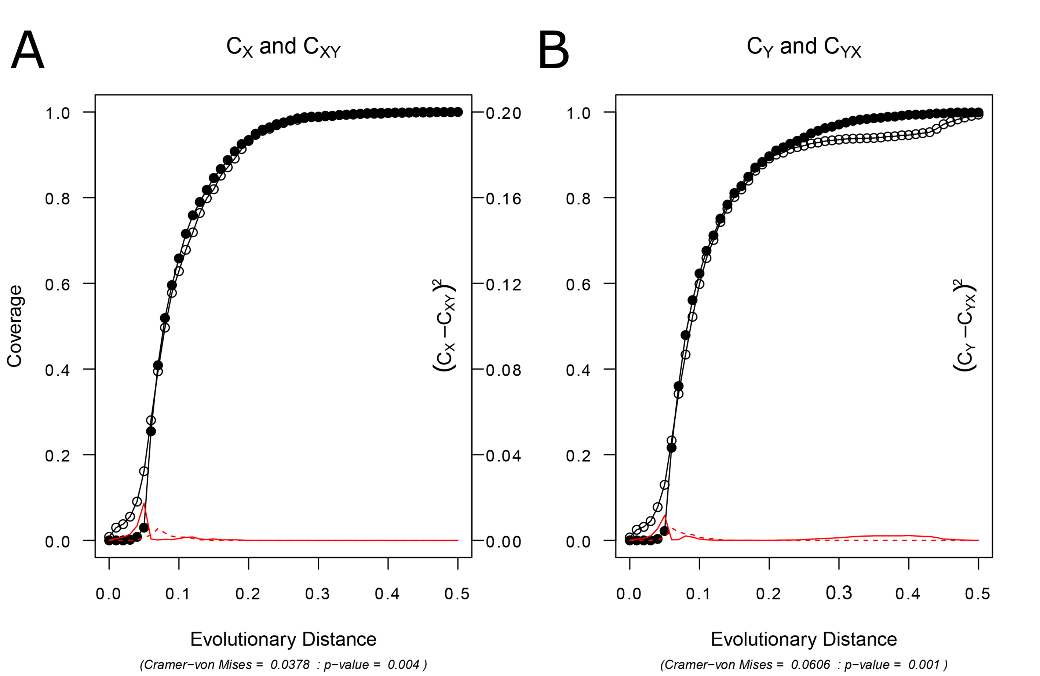


Fig B. Cramér von Mises-type statistic followed by a Monte Carlo test procedure comparing α-ISA driven community X with CDP driven community Y, comparing X with Y (A) and Y with X (B).


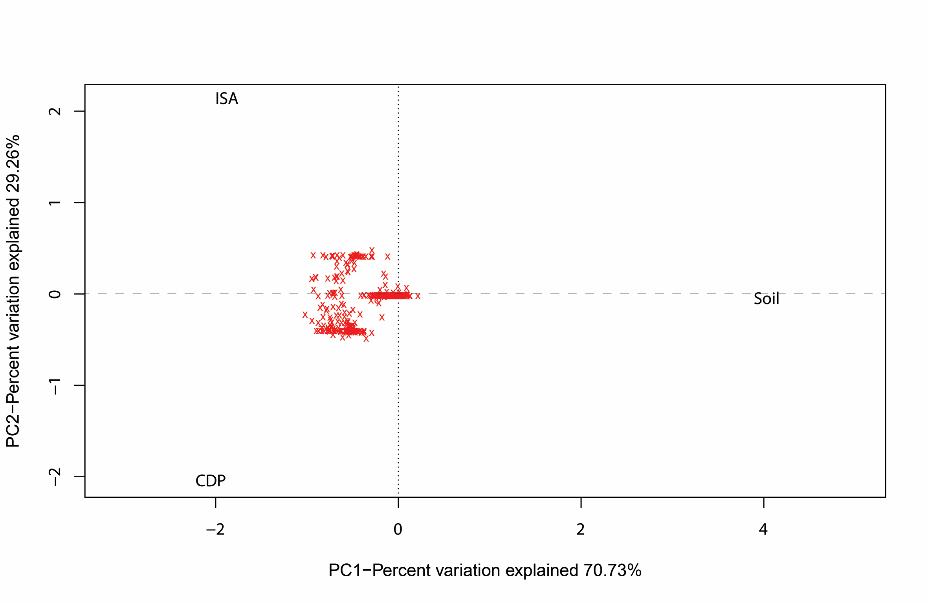


Fig C. Beta diversity analysis using Principal Component Analysis of original soil, ISA and CDP systems.
